# Supplementary material for: Effects of Microencapsulated Blend of Organic Acids and Essential Oils as a Feed Additive on Quality of Chicken Breast Meat
Source: Animals (Basel). 2020 Apr 7;10(4):640. doi: 10.3390/ani10040640 (PMC7222737; doi:10.3390/ani10040640)
Supplement: Supplementary file 1 [file animals-10-00640-s001.pdf]

Effects of Microencapsulated Blend of Organic Acids and Essential Oils as a Feed Additive on Quality of Chicken Breast Meat

Alessandro Stamilla [1], Nunziatina Russo [1], Antonino Messina [2], Carmine Spadaro [1], Antonio Natalello [1,*], Cinzia Caggia [1], Cinzia L. Randazzo [1] and Massimiliano Lanza [1]

[1] Dipartimento di Agricoltura, Alimentazione e Ambiente (Di3A), University of Catania, Via Valdisavoia, 5, 95123 Catania, Italy
[2] DVM consultant poultry specialists, via Cava Gucciardo Pirato, 12, 97015 Modica, Italy;
* Correspondence: antonio.natalello@unict.it; Tel.: +39-095- 4783473 (A.N.)

Supplementary Table S1. Effect of dietary treatment on microbial counts (expressed as $\log_{10}$ cfu/g) in raw meat during storage at refrigerated conditions.

Microbial groups	Day 0			Day 4			Day 7			Day 11		
	CON	AVI	*p-Value*	CON	AVI	*p-Value*	CON	AVI	*p-Value*	CON	AVI	*p-Value*
Enterobacteriaceae	3.98±0.03	4.03±0.81	0.876	6.39±1.11	6.78±0.21	0.364	7.71±1.30	8.32±0.65	0.274	9.68±0.27	9.63±0.37	0.768
Escherichia coli	1.39±2.41	1.51±2.62	0.928	2.45±2.20	2.31±2.05	0.899	2.57±2.23	2.75±2.38	0.885	1.67±2.89	<1	0.145
Faecal coliforms	1.55±2.69	2.54±2.38	0.468	2.69±2.45	3.27±1.11	0.568	2.61±2.27	2.58±2.26	0.975	7.30±0.22	7.18±0.53	0.583
Enterococcus spp.	1.00±1.73	1.90±1.85	0.354	2.86±2.52	5.81±0.14	0.009	6.35±0.38	5.83±0.80	0.134	7.19±0.10	6.58±0.65	0.028
Mesophilic bacteria	3.91±0.86	4.50±0.58	0.146	6.40±0.80	6.72±0.30	0.337	8.21±0.54	8.18±0.88	0.949	9.77±0.30	9.47±0.32	0.083
Psychrothrophic bacteria	3.34±0.75	1.86±1.64	0.050	6.18±0.24	5.83±0.62	0.189	7.04±0.04	7.08±0.11	0.405	8.27±0.07	8.31±0.03	0.192
Coagulase positive staphylococci	3.28±0.75	3.35±0.23	0.799	5.48±0.96	5.75±0.11	0.463	6.44±0.64	6.04±1.01	0.387	7.01±0.40	4.52±3.94	0.116
Coagulase negative staphylococci	3.09±0.18	3.03±0.55	0.766	4.56±0.05	4.13±0.11	0.000	6.50±0.20	5.59±0.02	0.748	6.20±0.17	6.79±0.80	0.086
Lactic Acid Bacteria	1.77±1.57	1.93±1.72	0.855	3.15±2.75	4.40±0.60	0.252	5.63±1.29	4.92±0.35	0.173	5.68±0.31	5.54±0.09	0.270
Lactococcus spp.	1.49±2.59	1.20±2.08	0.815	5.41±1.11	5.73±0.23	0.448	5.50±0.20	6.16±0.58	0.014	5.67±0.32	5.53±0.40	0.488
Yeasts/Moulds	3.96±0.91	3.59±0.93	0.462	5.94±1.06	6.47±0.10	0.198	8.81±0.53	8.48±0.41	0.202	9.87±0.17	9.95±0.18	0.413

Values are means ± standard error of the mean.
Control (CON) basal diet; basal diet + 0.5% organic acids and essential oils (AVI).
